# The impact of delays on maternal and neonatal outcomes in Ugandan public health facilities: the role of absenteeism

**DOI:** 10.1093/heapol/czw046

**Published:** 2016-05-03

**Authors:** Louise Ackers, Elena Ioannou, James Ackers-Johnson

**Affiliations:** ^1^Allerton Building, School of Nursing, Midwifery, Social Work and Social Sciences, University of Salford, Salford, M6 6PU, UK; ^2^Department of Obstetrics & Gynaecology, Homerton Hospital, Homerton Row, London E9 6SR; ^3^Allerton Building, School of Nursing, Midwifery, Social Work and Social Sciences, University of Salford, Salford, M6 6PU, UK

**Keywords:** Absenteeism, human resource management, low resource setting, maternal delays, maternal health, Uganda

## Abstract

Maternal mortality in low- and middle-income countries continues to remain high. The Ugandan Ministry of Health’s Strategic Plan suggests that little, if any, progress has been made in Uganda in terms of improvements in Maternal Health [Millennium Development Goal (MDG) 5] and, more specifically, in reducing maternal mortality. Furthermore, the UNDP report on the MDGs describes Uganda’s progress as ‘stagnant’. The importance of understanding the impact of delays on maternal and neonatal outcomes in low resource settings has been established for some time. Indeed, the ‘3-delays’ model has exposed the need for holistic multi-disciplinary approaches focused on systems change as much as clinical input. The model exposes the contribution of social factors shaping individual agency and care-seeking behaviour. It also identifies complex access issues which, when combined with the lack of timely and adequate care at referral facilities, contributes to extensive and damaging delays. It would be hard to find a piece of research on this topic that does not reference human resource factors or ‘staff shortages’ as a key component of this ‘puzzle’. Having said that, it is rare indeed to see these human resource factors explored in any detail. In the absence of detailed critique (implicit) ‘common sense’ presumptions prevail: namely that the economic conditions at national level lead to inadequacies in the supply of suitably qualified health professionals exacerbated by losses to international emigration. Eight years’ experience of action-research interventions in Uganda combining a range of methods has lead us to a rather stark conclusion: the single most important factor contributing to delays and associated adverse outcomes for mothers and babies in Uganda is the failure of doctors to be present at work during contracted hours. Failure to acknowledge and respond to this sensitive problem will ultimately undermine all other interventions including professional voluntarism which relies on local ‘co-presence’ to be effective. Important steps forward could be achieved within the current resource framework, if the political will existed. International NGOs have exacerbated this problem encouraging forms of internal ‘brain drain’ particularly among doctors. Arguably the system as it is rewards doctors for non-compliance resulting in massive resource inefficiencies.

Key Messages

- Human resource dynamics are the key to understanding maternal delays in low resource settings.
- Health worker absenteeism is a major contributory factor.
- Absenteeism is a particular concern amongst doctors.
- The failure of doctors to be present during their contracted hours directly contributes to delays and poor maternal and neonatal outcomes.
- Human resource management lies at the heart of this problem; policy attention to motivational factors and enforcement would significantly improve health system efficiency.

## Introduction

This article draws on research conducted within the frame of international health partnership interventions in the field of maternal and newborn health. The Liverpool–Mulago Partnership (LMP^1^) is one of many health partnerships twinning hospitals and universities in the UK with their counterparts in Uganda. Health Partnerships are often quite small, often charitable, organizations relying on individual volunteers (Ackers and Porter, 2011). In 2011 and with support from the Tropical Health Education Trust (THET), the authors set up an umbrella organization known as the Ugandan Maternal and Newborn Hub (the ‘HUB’) linking 10 health partnerships across Uganda.^2^ The HUB provided opportunities for improved coordination, team-working and evidence-based intervention. In 2012, the HUB received funding from THET’s Health Partnership Scheme (HPS) for a major intervention known as the ‘Sustainable Volunteering Project’ or ‘SVP’. The HPS is seen by THET as a mechanism to ‘harness UK health institutions and professionals in partnerships with developing country counterparts, and strengthen health systems through skills transfer and capacity development.’^3^ UK volunteers play a key role in this scheme which is designed to ‘leverage the knowledge and expertise of UK health professionals’. Echoing these objectives, the SVP identified two linked objectives:

1. To support evidence-based, holistic and sustainable ‘systems change’ through improved knowledge transfer, translation and impact.
2. To promote a more effective, sustainable and mutually beneficial approach to international professional volunteering (as the key ‘vector of change’).

Building on 4 years’ experience of volunteer deployment, the SVP has focused its energy on systems-changing interventions restoring functionality to referral health facilities with the aim of reducing congestion in referral hospitals and associated maternal delays. Since 2012 the SVP has deployed over 50 UK volunteers throughout the HUB in a diverse range of activities.^4^ The SVP and all related work in Uganda has been conceptualized first and foremost as ‘action-research’ supporting evidence-based policy development. In the context of exceptionally high and often increasing levels of maternal and newborn mortality, the work is part of a wider attempt to understand why ‘international development’ and particularly volunteering initiatives are failing to impact systems and how they may be improved.^5^

## Methods

The emphasis on process in a programme such as the SVP coupled with the paucity of reliable secondary data demanded an innovative and iterative multi-method approach. Building on many years’ experience of research on highly skilled mobilities and knowledge transfer processes, the evaluation strategy included a range of methods complementing and balancing each other through the process of triangulation (Iyer *et al.* 2013). As researchers we were acutely aware at the outset of the limitations of facility-generated secondary data. Accurate, reliable data on maternal and newborn health simply do not exist in Uganda. We therefore conducted a major benchmarking exercise across the 10 HUB facilities (including health centres and hospitals). This was an interactive process in itself and was as much about improving data collection and record keeping as it was about data capture; indeed the process included training of record keeping staff. These data should be regarded with caution (see below).^6^ As Gilson *et al.* note (2011) even in this ‘hard data’ context there is no single reality, no simple set of undisturbed facts and the data that we do see are essentially socially constructs.

The project has also used simple before-and-after testing schemes using Likert scales to assess learning and skills enhancement during formal training programmes. Capturing the impacts of volunteer engagement on health workers—and more specifically on behaviour change—is far more complex. We have utilized a range of measures including qualitative interviewing of volunteers, structured monthly reporting schedule for all volunteers and bi-annual workshops. Wherever possible volunteers have been interviewed at least three times (depending on their length of stay with interviews prior to, during and post-return). We have over 150^7^ verbatim transcripts drawn from all 10 HUB locations. Most of these have been conducted face-to-face in Uganda or the UK with some taking place via Skype. Where appropriate, email has also been used to discuss issues.

The research has also involved interviews and focus groups with Ugandan health workers, line managers and policy makers (about 50 to date). The authors have also spent many months in Ugandan health facilities and working with Uganda health workers in the UK. The project coordinator and manager each make regular visits (around 4 per year) ranging from 2 weeks to 5 months in duration. This intense observational research is recorded in project notes and diaries and is perhaps the most insightful of all of our methods. The qualitative material has been coded into a software package for qualitative analysis (NVIVO10) and subjected to inductive thematic analysis.^8^

In addition to this, volunteers have been encouraged, where appropriate, to develop specific audits to support contextualization and highly focused interventions. This has included audits on, for example, triage and early warning scoring systems, anti-biotic use and C-section rates. Building on detailed audit conducted by a Ugandan doctor on the causes and impact of delays or ‘decision-operation-intervals’ in the National Referral Hospital (Balikuddembe *et al.* 2009), a number of clinical volunteers have developed similar small scale studies focused on referring health centres. One of these studies conducted by a volunteer obstetrician is discussed in some detail in this paper. These audits are small scale and necessarily inherit the same problems with the accuracy of data and of medical records as the wider study.^9^

We have described the study as an example of action-research. It is necessarily iterative and as such we did not set out to achieve a specific sample size but have continued to spend time in Uganda interviewing and observing work in public health facilities and facilitating active workshops to encourage discussion around key issues. Indeed, it is through this iterative process that we have come to identify a key challenge that we believe is central to understanding both resistance to change in Ugandan health systems and the efficacy of professional voluntarism in low resource settings.

The article draws on this database with specific attention to research on human resource management informed by the work with Ugandan Health Workers and the audit of referrals conducted by the obstetric volunteer. It is interesting to note at this point that all of this research has attempted to go beyond narrow clinical assessments of individual obstetric cases to develop a deeper understanding of health systems and factors affecting health worker behaviour. Prolonged engagement with Ugandan health workers has enabled us to understand how, as Schaaf and Freedman suggest, ‘those who work on the ground, within struggling health systems, see and experience challenges that are all but ignored in the health literature’ (2013, p. 1).

The article is structured rather differently to many traditional papers with findings and discussion blended throughout in a more narrative style. This enables us to move from the general (figures on maternal mortality and its causes) through to more specific discussion of human resource factors.

## Maternal mortality in Uganda

**Figure 1.**
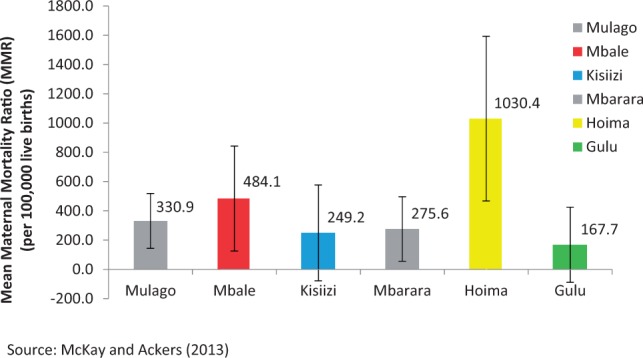
Mean maternal mortality ratios in referral hospitals 2011–12.

Figures on maternal mortality in Uganda vary considerably depending on the source. The World Health Organisation reports maternal mortality ratios (MMRs) in Uganda of 550 per 100 000 live births (WHO, 2010, P.26). The SVP benchmarking exercise (McKay and Ackers 2013) indicated wide variation between facilities in levels reported to the Ministry of Health. Perhaps of greater significance, it reiterated the very poor quality of reporting and records management in facilities resulting in significant underreporting. The figures for Hoima Regional Referral Hospital (figure 1) likely reflect improvements in records management following the intervention of a UK Health Partnership (the Hoima–Basingstoke Health Partnership) rather than a greater prevalence of mortality. Indeed, more detailed audit of case files by an SVP volunteer indicated levels in Mbale regional referral hospital of over 1000 (more than double reported levels).^10^

Maternal mortality in Uganda remains a serious and apparently intractable challenge despite the significant volume of international development investment. The key focus of the SVP has been to understand this challenge and identify ways of improving efficacy of volunteer interventions. This requires an in-depth understanding of causal factors.

## Understanding the underlying causes of maternal and neonatal mortality

Although the clinical conditions responsible for the final ending of a mother’s life remain all too consistent across low resource settings (haemorrhage, infection, eclampsia, obstructed labour, HIV/AIDS and unsafe abortion),^11^ the underlying causes demand more holistic and systems-focused analyses. The ‘3 delays model’ first espoused by Thaddeus and Maine (1990) emphasizes the temporal dimension of a complex sequence of individual decision-making and service failure marking the hazardous journeys women make along the ‘road to death’ (Filippi *et al.* 2005).

This emphasis on delays and the context within which women die helps to support evidence-based interventions focused on prevention and targeting the appropriate level of service delivery (Pacagnella *et al.* 2012). This certainly echoes our ethnographic experiences of working in the National Referral Hospital enabling us to make sense of the overwhelming congestion and atmosphere of ‘crisis management’ as women are admitted already near to death.^12^ Research conducted by Filippi *et al.* on near miss events in three African countries found that 83% of such cases were in a critical condition on arrival at the hospital (2005, p. 11). Similar findings are reported in studies of maternal mortality in rural Uganda (Kaye *et al.* 2011) and newborn deaths in Eastern Uganda (Waiswa *et al.* 2010).

According to the 3-delays model, the key to understanding the ‘maternal death puzzle’ lies in capturing the causes and outcomes associated with delays (Thorsen *et al.* 2012). It identifies a relatively linear continuum, ‘marking the interval between the onset of obstetric complication and its outcome’ (Thaddeus and Maine 1990, p. 1091). The first delay is associated with individual agency and the socio-economic factors shaping mothers’ care-seeking behaviour; the second, delays in reaching the facility and the third, delays in the provision of adequate care at the facility. Whilst the second delay is linked to delays associated with transport and access, in reality there may be a series of fraught journeys taking place as patients are (literally) bumped along dysfunctional referral systems. In that respect the second and third delays effectively merge as the lack of effective care at health centres results in further transport delays compounded by extensive waits in overwhelmed hospitals.

Iyer *et al.* (2013) present an alternative ‘missed opportunity’ approach to the 3-delays model critiquing the implied linearity and embracing more effectively the power dynamics involved. More specifically, they propose the use of social autopsy to capture the multiple and often conflicting narratives of actors to challenge the ‘bio medical ‘causes of death. We would very much subscribe to this approach. The well-being of mothers ultimately stems from their status in the family and community and the most effective interventions lie in female empowerment and improved family planning. However, clinical interventions by expatriate volunteers have tended to focus on the large referral facilities, perhaps reflecting the interests and the ‘knowledge paradigms’ (Gilson *et al.* 2011, p. 1) of the clinicians involved. Indeed, a central focus of much HP activity has been on emergency obstetric care rather than preventive interventions. The deployment of volunteer doctors through the SVP has sought to take a step back from this and address referrals into these large hospitals from those health centres [Health Centre IV (HCIVs)] in Uganda that are, in theory at least, designated to provide comprehensive maternity services including emergency obstetric care.^13^

The Ugandan Ministry of Health’s Strategic Plan identifies the lack of health centre facilities providing emergency obstetric care as a key challenge. This problem is linked specifically in the report to the ‘weakness of referral systems’ (2010, p. 36). The objective of the previous Health Strategy (HSSPII) was to ‘ensure a network of functional, efficient and sustainable health infrastructure for effective health service delivery closer to the population’ (p. 19). This included the construction and refurbishment of operating theatres and maternity wards. However the Annual Health Sector Performance report suggests that ‘most facilities and equipment are in a state of disrepair’ (p. 19) and calls for the Government to, ‘mobilise resources in order to increase the functionality of HCIVs from 5 to 50% and create a fully functional national referral system’ (p. 20). In the Ugandan health system, HCIV facilities should be the closest facility to mothers providing 24-h emergency obstetric care (including caesarean sections). However, a national assessment found that only 3% of these facilities did so (UNDP 2013, p. 25).

This situation reflects weaknesses in physical infrastructure and resource (equipment, consumables, power or blood) combined with a ubiquitous ‘human resource crisis’. This ‘crisis’ remains under specified with vague references to an overall lack of personnel and/or lack of necessary training and skills (Thorsen *et al.* 2012). Indeed, it is hard to find a study that does not refer to the lack of skilled personnel in facilities across low- and middle-income countries (LMICs) as a major factor. However, the reader is often left wondering what lies behind this situation.

Generic reference to ‘staff shortages’ tells us very little about the situation on the ground and the reasons behind shortages. It is easy to assume that the problem is simply down to an overall lack of qualified personnel perhaps due to inadequate training capacity and/or international brain drain (emigration). Both of these are important contributory factors. Certainly recruitment, especially in lower level facilities and rural areas, is an immense challenge: in 2008, Ministry of Health (MOH) figures suggested that only 51% of approved positions at national level were filled with more rural/peripheral locations faring worst.

Research conducted by three Ugandan specialists (Balikuddembe *et al.* 2009) assessed the length, impacts and causes of delays in conducting emergency caesareans in Mulago National Referral Hospital. Although the lack of theatre space was listed as a factor contributing to delay in nearly all cases, the study highlighted major problems in human resources and the presence of staff (see table 1):

**Table 1.** Common factors determining the decision-operation-interval (Mulago Hospital, Uganda)

Rank	Factor	Mean time lost (minutes), *n* = 351^a^	% Mothers affected
1	No theatre space	366.5	94.0
2	Shift change-over period	26.1	22.2
3	Instruments not ready	15.1	21.4
4	Surgeon on a break	13.7	24.5
5	Anaesthetist on a break	11.7	6.8
6	Theatre staff on a break	6.4	13.7
7	Some theatre staff not arrived	5.1	12.5
8	Linen not ready	3.7	7.7
9	Irregular patient drug dosing	3.3	1.1
10	Anaesthetist not arrived	2.8	4.0
11	No theatre sundries	2.1	5.7
12	Patient unstable	1.7	2.3
13	Patient not seen on ward	1.6	0.6
14	Lack of i.v. fluids	0.5	2.0
15	Patient not consented	0.4	0.6
16	Surgeon not arrived	0.3	0.6

Source: Balikuddembe et al. (2009).

^a^Assume all 351 participants’ DOI could be affected by all the factors.

Asked to explain the reasons for the human resource crisis, an experienced Ugandan health professional replies:

To start with really they don’t have enough people trained to fill all the possible positions. I know that almost all the big hospitals are advertising positions for doctors and nurses. I also know lots of doctors who don’t want to practice as doctors because they can work as consultants in an NGO. They usually go to American funders, they basically look around everywhere for anyone interested in funding their opportunities. People are now trying to go for project jobs. One good thing that people have realised now is you can work in a government institution because there you are guaranteed more like a lifetime job, at the same time there are so many projects that come into the government institutions and help people kind of top up their salaries in one way or another’. [UHP10]

The respondent identifies a number of contributory factors. In the first instance, he indicates problems in initial supply exacerbated by the haemorrhaging of doctors from clinical work into (usually non-clinical) positions in NGOs. Others strategically seek to combine ‘project’ work with their full-time public roles (contributing to absenteeism).

The respondent later refers to the problems of international brain drain suggesting that many Uganda doctors are looking for better paid work across the border in Rwanda, for example. But this is compounded by the often more damaging effects of ‘internal brain drain’ (Ackers and Gill (2008)). In Uganda, this manifests itself in many doctors studying for Masters Degrees in either Business Administration (MBA) or Public Health (MPH) positioning themselves to work in NGOs in managerial positions.

Linked to the above, remuneration is a key factor affecting the presence of doctors in public health facilities. At the present time private work (‘moonlighting’) is, in theory, illegal and banned. In practice, it is endemic. To some extent, this represents a natural and entirely logical response to low pay. The following Ugandan health worker explains both the need for salary augmentation and the importance of holding a position in the public sector to this process:

Most doctors working in the private sector are working for themselves simply because they need to make a bit of extra money and that way they can even negotiate to take some of the patients from the public hospital to their private hospitals. [UHP21]

In addition to the low level of pay, serious administrative problems in many districts mean that healthcare staff are not paid at all for months:

Right now they are not paying them enough and it doesn’t come on time. I know people who don’t get paid for six months and they expect them to carry on smiling, offering the best services they can when their landlords are chucking them out because they don’t have money to pay. [V39]

The respondent had personal experience having waited for over 6 months to be paid (in this case by a university).

Remuneration remains a major problem but it is never the only factor (Dieleman *et al.* 2006; Garcia-Prado and Chawla 2006; Mathauer and Imhoff 2006; Mangham and Hanson 2008; Willis-Shattuck et al. 2008; Mbindyo et al. 2009; Stringhini *et al.* 2009). And, it is not at all clear that a recent MOH initiative to significantly increase the pay of doctors in HCIVs (to 2.4 million per month—around £500) has translated into (any) increased presence on the ground (for further discussion, see Ackers and Ackers-Johnson 2016).

According to the MOH, staff shortages are compounded by ‘high rates of absenteeism and rampant dualism’ (Ministry of Health 2010, p. 20).^14^ In a rare study focused specifically on the absenteeism of health workers Garcia-Prado and Chawla (2006, p. 92) cite WHO statistics indicating absenteeism rates of 35% in Uganda. A senior manager of a Ugandan Health District reported (UHP49) much higher genuine rates of absenteeism suggesting that during a recent personal visit over 65% of his staff were ‘on “offs” ’ at any point in time. This certainly confirms our experiences as ethnographic researchers and is likely to significantly over-estimate the presence of doctors. In the following focus group with Ugandan mid-wives and doctors, respondents were asked about health worker absenteeism. They talked at length about mid-wives and nurses but did not mention doctors:

Interviewer: You haven’t mentioned doctors at all?

(Laughter between everyone)

Respondent 1 (midwife): Oh, sometimes we forget about them because most of the time we are on our own. You can take a week without seeing a doctor so we end up not counting them among our staff.

Respondent 2 (doctor): Especially on a night, you never see them there (at the health centre).

Respondent 1: Even during the day like most of the time.

Interviewer: How often would you say a doctor would come to the facility in a typical month?

Respondent 1: The medical officers have the rest of this centre to cover too so maternity will see them only if there is any problem. So they come for two hours three times a week but that’s for the whole centre, the other wards as well.

Respondent 2: Yes, like two times a week, sometimes once but most of that time even when they’re on [duty] someone will not come to review the mothers.

Interviewer: What would happen if a mother needs a caesarean? Would you call the doctor?

Respondent 1: Initially they told us we should call before [referring] but every time you call that doctor he is going to tell the same thing: ‘I’m not around, you refer’. And you use your own judgement but sometimes you follow protocol, because if anything happens … you call that doctor for the sake of calling.

Interviewer: Just going through the process?

Respondent 2: But you know he’s not going to come [FGUK04]

Whilst physical infrastructure and supplies of consumables continue to contribute to facility down-time, it is clear that human resource factors are a major contributing factor. And, in many respects the former are used as excuses to conceal the latter. One facility manager explains that, at the time of interview, there were few other factors restricting the use of theatre:

Now we have constant power—the power is there. We had issues of water now they’ve stabilized. Now water is flowing; the issue of drugs we have sourced drugs.

Interviewer: But the doctors are still not here?

No, they don’t even come and you have to keep calling. You will call the whole day and some will even leave their phone off. [Referring to a list of referrals] Take this [referral] is for a ‘big baby’ but this is a doctor, an obstetrician. [I asked] when you referred this case, why wouldn’t you enter into theatre? We are making many referrals to [the Hospital] and they are complaining. [The doctors] are very jumpy, they work here and there. So, we had a meeting and one doctor was very furious about [the decision to question referrals]. I said, no this is what is on the ground; we want people to work. And the reason [they give] is there’s no resting room. There may be issues of transport (i.e. the doctors’ personal transport), but there’s also negligence. [UHP32]

It is not simply that doctors work very few hours but the unpredictability of their presence and the absolute unwillingness to commit to any set hours that impacts services. In several HCIV facilities, we have attempted to institute elective C-section lists in the hopes that this would enable us to be sure that UK volunteers could work alongside Ugandan doctors but also to prevent emergencies arising. This has proved absolutely impossible. It is no surprise, in this situation, that the overwhelming majority of caesarean sections in Uganda is undertaken as emergencies allowing complications to develop and outcomes to worsen. A recent audit in Mulago Hospital (Acen 2015) found that of 200 C-sections undertaken in September–November 2014, 184 (92%) were emergency sections. This stands in stark contrast to the UK where emergency caesareans are reducing in proportion (NHS 2014).

Most of the 15 or so obstetric volunteers have attempted at some point to introduce elective lists: all have failed. Asked about the prevalence of elective C-sections an experienced British mid-wife (SVP volunteer) who worked for 4 years across a number of health centres remarked:

What is an elective? I haven’t seen any here at HCIVs—possibly a few private ones.^15^ [V31]

Accommodation is a serious issue (as noted above) but it is not a panacea especially when it comes to doctors. The LMP funded a doctor’s overnight room in one facility but it has yet to be utilized. Where we have provided an overnight room for mid-wives (in another facility) we have achieved and sustained 24/7 working. However, in one of the health centres we are involved with where doctors benefit from the provision of dedicated (family) housing on site, this has not improved their presence:

Caesarean section mothers operated on Thursday or Friday are generally not reviewed by a doctor over the weekend. One mother operated on for obstructed labour whose baby died during delivery had a serious wound infection, pyrexia and tachycardia and pleaded (4 days later) for me to help her. (SVP volunteer V31 report to District)^16^

Whilst absenteeism and poor time-keeping is an endemic problem amongst all cadres in Uganda, the situation is most acute when it comes to doctors. ‘In-charge’ doctors (senior medical officers appointed as facility managers) are often the worst offenders setting a very poor example to medical officers in their facilities and failing to observe and enforce contractual terms.^17^ As the following respondent suggests, many if not most of the doctors in these leadership positions do not do any clinical work in the public facility they preside over:

Most of the (in-charge doctors), if you really look at them, want to do administrative work actually, they want to sit in the office—they sign out the PHC (primary health care) fund. It’s at their discretion to spend it so…. And of course sometimes there’s corruption, outright corruption.

Interviewer: So really what they’re doing is administration but not leadership.

Leadership requires you to be around, you can’t let people run the place when you’re not there. Leadership needs your presence, so you know the fact that [the in-charge doctors] are not always there, it’s difficult. [UHP29]

Where in-charges are nurses, mid-wives or administrators, they have very limited ability to hold doctors to rotas:

[Enforcement] is a problem. Doctors don’t want to be accountable to someone ‘below’ them. They don’t want someone, even if someone has a degree but they’re not a doctor, to keep instructing them. [UHP29]

This problem of enforcement seems to stem from higher levels with District Health Officers seemingly powerless to sanction poor behaviour:

I think particularly in the health department they are still intimidated by doctors which is a bit surprising. It goes hand in hand with accountability because if I know I am accountable for something going missing and if it goes missing then something will be done to me, in terms of discipline then of course I will behave differently. I wouldn’t want to be found doing something on the wrong side of the law because I know that there is action that is going to be taken against me. But because here people don’t see anything being done then they can do lots of things. [V39]

Interestingly he goes on to contrast the quite severe enforcement of anti-theft measures recently instituted during building works at Mulago hospital with the ‘blind eye’ approach to absenteeism suggesting that this is not a general problem of enforcement in Uganda but one connected specifically with human resource management culture:

I can give an example, in Mulago they are renovating and if you are caught taking something out of the hospital without proper papers you will be dismissed indefinitely. You can steal something worth 10,000 shillings (£2) and in the newspapers you will be regarded as a thief. Now people know where they stand: they can choose to disobey the law but they know that if you walk on the grass verge (in Kampala) they will get fined 100,000 shillings.

The following section reports on an audit conducted by an SVP volunteer as part of an intervention designed to reduce referrals to the national referral hospital.

## Auditing referrals—a case study

Whilst the research on maternal delays points to the human resource crisis in public healthcare, the relationship between absenteeism and maternal outcomes is rarely studied explicitly. On-going engagement of SVP volunteers with health workers in one health Centre indicated serious concerns. It was in this context that [the obstetric volunteer] undertook an audit focused on the impact of absenteeism.

In 2014, at the specific request of Kampala City Council Authority and with the involvement of a multi-disciplinary team of SVP volunteers working with local staff we were able to commence deliveries in a facility that had never been operational since its construction in 2008. Deliveries commenced within a week and have risen to over 600 per month (Ackers 2014b). However, large numbers of referrals continue to be made to Mulago hospital contributing to congestion (in a facility delivering 33 000 babies a year) and significant delays. Many of these referrals are due to the failure of doctors on rotas to be present. The Health Centre is different to many other HCIVs in that it is purpose built, has good general infrastructure, benefits from the quite unusual presence of a dedicated ambulance and is only about 4 km from Mulago Hospital. As a project we were delighted at the rapid growth in deliveries over the first year. However, we found it exceptionally difficult to get theatre functioning due to the failure of the four doctors employed there to report for work with any degree of consistency or predictability. This meant that, in practice, the facility has been functioning more as a Health Centre III (mid-wifery lead unit).

**Figure 2.**
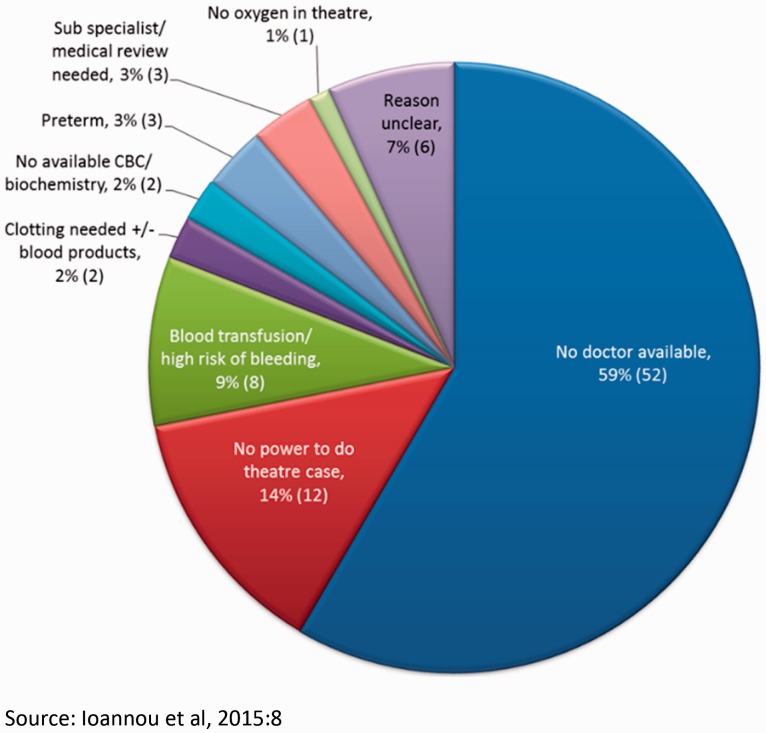
Primary reasons for referral (*n* = 89).

**Figure 3.**
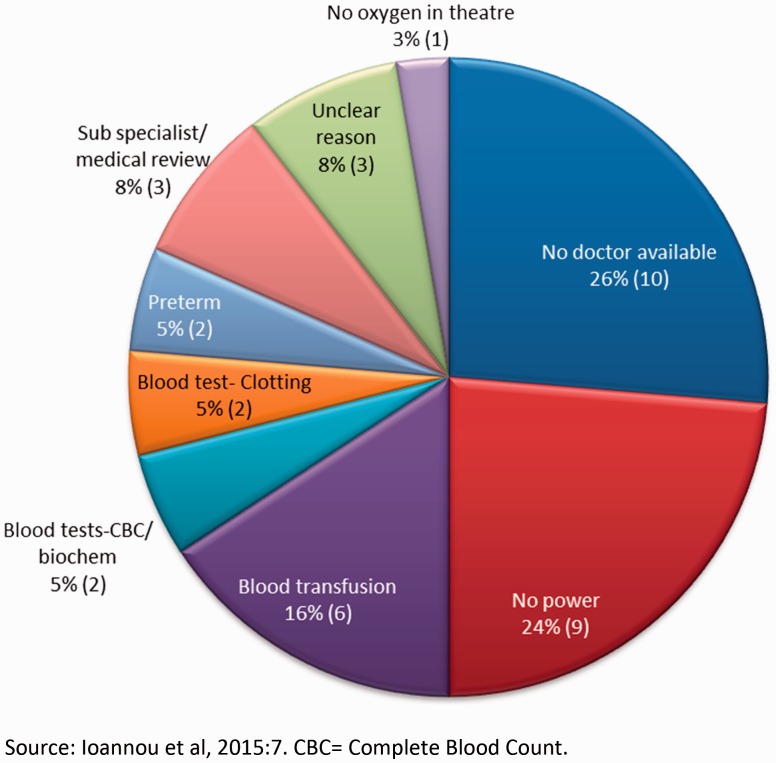
Reasons for referrals between the hours of 08:00 and 17:00.

During her placement the obstetrician observed that, ‘when [the Health Centre] runs well patients are normally seen and treated within a few hours of a problem arising’; however, referring women increases time delays to their treatment especially when referring to an already congested facility. Minimizing such referrals and delays should improve maternal and neonatal outcomes. The audit took place over a 2-month period (December 2014–31 January 2015) and attempted to audit all obstetric and gynaecological referrals during that time frame. One of the major limitations of the study was the poor quality of patient records and failure in many cases to accurately record times and reasons for referral. The results are presented in detail in Ackers (2015). This article will focus on obstetric referrals. Figure 2 presents data on the primary reasons for referrals: 59% of referrals were due to the failure of local doctors to be present during their contracted hours:

Figure 3 presents the timing of referrals; whilst many of these referrals were made in the evenings and at night the problem was also present during the day:

Bharmal and Bell-Webb’s audit in another region (2015) identified similar patterns. Here, over 78% of referrals to the Regional Referral Hospital were not reviewed by the resident doctors; many of these were during the day and at weekends. It is relevant to point out here that two doctors are accommodated on site in this facility.

**Figure 4.**
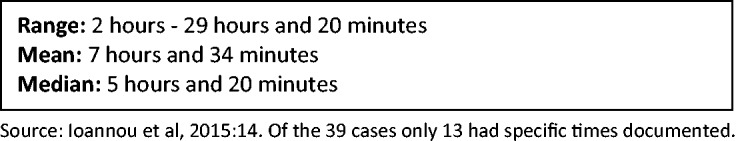
Time from decision to transfer to caesarean section at Mulago Hospital.

Figure 4 reviews the delays associated with referrals in 39 obstetric cases for which data were available. Patients at the Kampala Health Centre are fortunate to benefit from the presence of a fully functioning (new) ambulance which is highly unusual in an HCIV facility in Uganda. Although records are very poor in this area, where times are recorded the time taken for the ambulance to arrive at the Health Centre was on average 40 min and the journey took, on average, 45 min.^18^

Bharmal and Bell-Webb (2015) also report extensive delays. In this context, the distance between facilities is about 33 km with the average (mean) time between referral decision and arrival at the referral hospital being 6 h.^19^ Taking delays incurred following arrival at the hospital, mothers faced a mean delay of just under 21 h between the original referral decision and actual delivery (with a median of 23 h). If we take only those mothers who went on to have a caesarean section, the mean delivery interval time was over 22 h.

The volunteer obstetrician presented detailed notes focusing on cases with poor outcomes. As expected in some cases the assessment supports the decision to refer and/or the lack of a causal relationship between the presence of doctors and patient outcomes. However, in nine cases the absence of a doctor appears to contribute directly to poor outcomes for mothers and babies. For brevity, we present two of these cases to illustrate the relationship between medical presence and maternal/neonatal outcomes:

### Case 1: Maternal death and macerated still birth

This 19-year-old woman with a twin pregnancy was referred for caesarean section at 16.30 on a Saturday due to slow progress. It is unclear why she was referred at this time as a doctor should have been on site. There was no evidence that a doctor had seen the patient prior to referral. She was subsequently seen by a doctor in Mulago Hospital at 17.55 and the decision was made for caesarean section. At 11.00 the next day she was fully dilated and membranes were felt and ruptured. At this point, the liquor was foul smelling and there were signs of obstruction. The operation was not performed until 21.50, 29 h and 20 min after referral. The first twin was born alive. However, the second twin was a macerated still birth and pus was found in the uterus. The mother became very unwell and 2 days later was admitted to the high dependency unit with severe sepsis. She died 2 days later.

Clearly, the long delay, which is not uncommon in Mulago (for reasons discussed above), contributed to this woman’s death. However, had a doctor been present and her case managed effectively at the Health Centre this could have been avoided.

There is no reason why this patient could not have been delivered 29 h earlier at the health centre. The delay is very likely to have contributed to the degree of sepsis and hence the final outcome. This was an avoidable referral resulting in long delays.

### Case 2: Fresh still birth

This 28-year-old woman was referred at 21.35 with a diagnosis of cord prolapse. There was no doctor on site to review the case. She was referred to Mulago (no timings available) and seen by a doctor at 23.25 when she was 6 cm dilated and a foetal heart documented to be present and a normal rate. The plan was for caesarean section but there were no theatre linens available. At mid-night she was scanned and an intrauterine foetal death confirmed.

Could this referral have been avoided?

This outcome could have been avoided if a caesarean had taken place in the facility within 2 h.

Ioannou *et al.* conclude, ‘on the basis of this audit that 9 cases (10% of total) resulted in significant negative outcomes: at least 4 of these outcomes were very likely to have been avoided if doctors were present between the hours of 17.00 and 08.00 am and the patient was delivered earlier at the health centre’ (2015, p. 21).

As well as echoing serious concerns about record keeping (documentation) that made the audits very difficult, Bharmal and Bell-Webb identify the problem of ‘self-referrals’ with many patients deliberately by-passing referral units that they know are not functioning adequately and referring themselves directly to the Regional Referral Hospital. If patients are aware that staff are not present on a reliable and 24-h basis, many will literally vote with their feet. It is hard to gauge with any accuracy the prevalence of this phenomenon. Analysis of 10 000 cases mapping the villages mothers came from in Mulago Hospital provided some indication of self-referrals (Ackers *et al.* 2010). The same report showed that of the 211 maternal deaths recorded in Mulago Hospital between April 2006 and November 2009, 19.4% were self-referrals.

As noted above, this audit illustrates not only the serious impact of delays but also the paucity of accurate facility-based data in Uganda and the difficulties in tracking cases. And this is not simply a technical/capacity issue. Data are often deliberately interfered with by doctors involved in corrupt practices.^20^ The audit is presented here as indicative of the situation in all of the HUB public health facilities. It is by no means unique. A volunteer obstetrician reported a very similar sequence of events over the space of 1 week affecting referrals from a HCIV facility (in another region) into a regional referral hospital. This case highlights the impact of absenteeism of senior doctors not only on UK volunteers (who, according to our co-presence model, should withdraw from clinical practice in such scenarios)^21^ but also on junior Ugandan doctors many of whom are receiving little if any supervision and working dangerously long hours:

The obstetric department at [referral hospital] is overwhelmed with the patient load. The interns currently staffing the unit are working 100–150 hours a week. The interns I have encountered are without exception highly professional and enthusiastic about patient care and quality improvement. […] However, I know personally of three occasions where consultants were contacted for help with complex cases and did not attend. On XXX (a Sunday) I was called by one of the intern doctors requesting help with a complex ruptured uterus. Unfortunately I was in [another town] so I could not attend. He informed me that he had attempted to contact all the (4) consultants but that none of them responded. In the end one of the interns who previously worked in obstetrics and gynaecology and is now working in surgery came to assist with the repair.

[One week later] I attended a Caesarean section in the afternoon to receive the baby while awaiting theatre space to begin one of three Caesarean sections waiting to be done. The intern performing the section found that the uterus inverted when he attempted controlled cord traction delivery of the placenta due to placenta accreta. Together with the medical officer, the three of us proceeded to perform a subtotal hysterectomy. On this occasion the consultant on duty was contacted and asked to attend but informed us to proceed without him.

[The following day] a patient was transferred from [HCIV] with a ruptured uterus and arrived just after 6 am. The patient was in theatre at 8.30, however due to the anaesthetist arriving late the procedure was not commenced until 10 am. The foetus was stillborn and a significant rupture extending into the left broad ligament and down towards the cervix was identified. Two consultants were contacted and asked for help, however neither attended. The procedure was performed by the intern and the medical officer.

I have observed that a high number of the referrals into [the regional referral hospital] are coming from [a particular HCIV] which is a source of great frustration to the staff at FPRRH. [Over the past month another HCIV facility] referred half as many patients as the previous month. I believe this reflects the presence of a doctor at the centre on a daily basis. [V52]

This case illustrates the kind of scenarios that are experienced on a daily basis by UK volunteers working in Uganda public health facilities across the HUB and the effects this has on patients, peer workers and UK volunteers.

## Conclusions

This article has presented research evidencing the impact of absenteeism on services and patient outcomes in Uganda. As such it represents a highly contextualized ‘thick description’. Whilst the results of such a study cannot be said to be statistically generalizable, the HUB environment enables us to combine and compare case studies within the Ugandan national context presenting opportunities for important theoretical insights that we believe apply across the Ugandan ‘public’ health system and, potentially, other low resource settings. Gilson *et al.* suggest that this type of work with a strong and essential emphasis on the dynamics of context and triangulation ‘challenge the HPSR community to think more deeply about how to support policy and system change through the generation of “middle range” theories’. (2011, p. 4)

The failure to develop an effective human resource management system in Uganda capable of both incentivizing and enforcing adherence to contractual terms is responsible for serious resource inefficiencies. Whilst this problem is evident across all cadres from cleaners to specialists, doctors possess a degree of autonomy that enables them to avoid compliance with impunity. As doctors are also, in most cases, in leadership (management) positions this removes the opportunity for effective role modelling for other cadres. Where the overwhelming majority of doctors fails to present themselves for work with any degree of regularity or predictability, the costs to the public purse are enormous. Improved human resource management could release significant funds to augment the salaries of those staff that do present for work as well as creating the environment for the development of effective team-working.

The planned decentralization of health systems management giving greater autonomy to Health Districts and potentially devolving power to in-charges has the potential to iron out some of the serious delays in paying health workers and also to increase accountability in human resource management and enforcement of employment contracts. At the present time, salary augmentation through private working is essential to persuade doctors to work in the public sector. We would urge the Ministry of Health to consider the possibility of openly permitting a degree of private working within the frame of existing contracts and subject to stringent accountability and enforcement mechanisms. International NGOs and development organizations should also attend to the externality effects associated with interventions in particular the distorting effects on local labour markets.

## Notes

1. www.liverpoolmulagopartnership.org.

2. These include the national referral hospital, five regional referral hospitals, one mission hospital and a large number of feeder health centres. For details, see www.liverpoolmulagopartnership.org.

3. http://www.thet.org/wp-content/uploads/2011/09/HPS-Volunteering-Grant.

4. The SVP Annual Report 2013 provides full details of the project and its objectives. It can be found in the ‘documents’ section of the LMP website alongside a series of research and policy reports.

5. This concern forms the focus of a book based on the project (Ackers and Ackers-Johnson 2016).

6. Full details are reported in McKay and Ackers (2013).

7. The numbers cited here are constantly increasing as we continue to deploy volunteers and assess impacts.

8. We have used the prefix UHW to identify Ugandan Health Worker respondents; FG for focus groups and V for SVP volunteers.

9. The epistemological approach underpinning this research and the methodological implications of that are discussed in greater detail in Ackers and Ackers-Johnson (2016).

10. These figures only record deaths in the facility and thus miss cases where mothers die in the community.

11. Detailed data on causes of deaths can be found in McKay and Ackers (2013).

12. The health centres involved in the SVP benchmarking report reported no maternal deaths. Sadly this should not be seen as indicative of the quality of care in that facility but a practice of transferring mothers even when they are near to death. It is not unusual for such mothers to die before or on arrival.

13. Placing volunteer doctors in midwifery lead Health Centre 3 facilities has proved unattractive to SVP volunteers although many have embraced these as an aspect of their systems-focused work. The authors are currently involved in a follow-up project evaluating the impact of professional volunteers on the volunteers themselves and the NHS (http://www.salford.ac.uk/nmsw/research/research-projects/move).

14. Absenteeism presents specific challenges for a programme such as the SVP committed to the principle of ‘co-presence’ and avoiding labour substitution wherever possible. Put simply, where Ugandan staff are regularly absent and the risk of lone working is high, we are unable to place professional volunteers (list of authors’ references).

15. Doctors attached to HCIVs often bring their private patients into the facilities to operate on privately. The incidence of this is difficult to know as records are often (perhaps deliberately) imprecise.

16. In a pilot project, our charity has recently constructed purpose built accommodation for a Ugandan obstetrician in order to enable a regional referral hospital to attract a suitable candidate (they were faced with the prospect of having no obstetrician present at all which also meant we could not place long-term volunteers there). We have attempted to link conditionality principles to occupancy to ensure that the doctor works to his employment contract. We are currently monitoring the project. This work has been undertaken in conjunction with a sister charity ‘One Brick at a Time’ (OBAAT). For further details, see www.lmpcharity.org.

17. This may reflect wider issues associated with the relative autonomy and power of the medical profession (Sheikh and Porter 2011a,b).

18. Transport delays play a much larger role in other facilities (Aldrich 2014).

19. This region benefits from an unusually good ambulance service supported by Baylor (an American NGO).

20. There is insufficient scope in this article to go into detail on this but see Ackers and Ackers-Johnson (2016).

21. Co-presence is an operational concept central to the SVP model and requires that UK doctors are never involved in lone working (gap-filling). For further details, see Ackers and Ackers-Johnson (2016).
